# Meningitis and a Febrile Vomiting illness Caused by Echovirus Type 4, Northern Territory, Australia

**DOI:** 10.3201/eid1601.081519

**Published:** 2010-01

**Authors:** Peter G. Markey, Joshua S. Davis, Gerry B. Harnett, Simon H. Williams, David J. Speers

**Affiliations:** Centre for Disease Control, Darwin, Northern Territory, Australia (P.G. Markey); Royal Darwin Hospital, Darwin (J.S. Davis); Queen Elizabeth II Medical Centre, Nedlands, Western Australia, Australia (G.B. Harnett, S.H. Williams, D.J. Speers)

**Keywords:** Echovirus 4 infection, enterovirus, viral meningitis, viruses, bacteria, Australia, research

## Abstract

In July 2007, a cluster of meningitis cases caused by an echovirus 4 strain was detected in 1 indigenous community in the Top End of the Northern Territory of Australia. Illness was characterized by fever, vomiting, and headache. Over the next 4 months, additional cases of meningitis and the fever and vomiting syndrome emerged in other indigenous communities and subsequently in the major urban center of Darwin. We describe the epidemiology of 95 laboratory-confirmed meningitis cases and conclude that the epidemic fever and vomiting syndrome was caused by the same enterovirus. Nucleotide sequencing of the whole genome verified this enterovirus (AUS250G) as a strain of echovirus type 4. Viral protein 1 nucleotide sequencing demonstrated 96% homology with an echovirus 4 strain responsible for a large outbreak of meningitis in the Yanbian Prefecture of China in 1996.

## Abstract 2

A strain that emerged in July 2007 caused laboratory-confirmed meningitis.

Enteroviruses are among the most common human viral pathogens. Recent reports from the United States have documented >50 serotypes of enterovirus causing illness in humans (*1*). Illness syndromes with established causal links to enteroviruses include acute hemorrhagic conjunctivitis; viral meningitis; hand, foot, and mouth disease; and acute ascending paralysis (including poliomyelitis). In July 2007, a disease control unit in the Northern Territory of Australia reported a cluster of viral meningitis cases in a nearby community. Nucleic acid testing of the cerebrospinal fluid (CSF) of these patients detected an enterovirus.

The Northern Territory of Australia has a population of 210,000 living in an area of 1.35 million km^2^; the climate varies from desert and semiarid in the south to subtropical in the north. The northern part, known as the Top End, is characterized by several small urban centers and many small scattered indigenous communities with populations of 300–2,000. Darwin (population 100,000) is the major urban center. Approximately 30% of the population of the Northern Territory consists of indigenous Australians.

This cluster of meningitis cases coincided with an outbreak of another viral illness in the same and nearby communities; the illness affected mainly children and was characterized by fever, vomiting, and headache. During the next 4 months this epidemic febrile vomiting syndrome (EFVS) was reported in multiple communities in the northern part of the Northern Territory and eventually in Darwin. During the same period, clusters of viral meningitis were also being reported in some of the communities experiencing the EFVS.

We hypothesize that the cluster of viral meningitis cases and the EFVS were different manifestations of the same infection, caused by a strain of echovirus type 4 (E4) virus. This enterovirus was closely related to 2 E4 strains that caused a large outbreak of viral meningitis in the Yanbian prefecture of China in 1996 (*2*); ≈5,000 cases from a population of 2.16 million were identified.

## Methods

### Patients

A case of acute E4 enteroviral illness was defined as the detection, in 2007 in a resident of the Northern Territory, either of E4 in a CSF specimen or E4 in samples from another site during an illness characterized by fever and severe headache. Infants were children aged <1 year; children (including infants), <15 years of age; and adults, >15 years. Cases were included in our study only if specimens were collected in 2007 and samples from patients had a positive PCR or culture for E4 at the reference laboratory. A questionnaire was developed and details of cases were collected by a review of hospital case notes and, where possible, by telephone interviews with case-patients. The questionnaire documented clinical symptoms and signs together with laboratory results and risk factors, such as potential occupational exposure, child care, institutional exposure, or illness in the immediate family. Duration of illness was identified by discussion with the case-patient or, if the case-patient could not be contacted, was defined as the difference between date of symptom onset according to the medical record and date of hospital discharge.

The spread of the EFVS was investigated by asking senior clinic staff at all the remote community health centers in the regions affected about the local presence of a recent epidemic of fever, vomiting, and headache. If staff recalled such an epidemic, details were recorded about its timing, number of case-patients seen in the community health center, and the proportion of case-patients who were children. These interviews were all conducted within 2 months after the outbreak. Attack rates were calculated by using these estimates and the population of each community according to 2006 census data from the Australian Bureau of Statistics (*3*).

We collated data in Microsoft Excel 2000 (Redmond, WA, USA) and performed statistical analysis using STATA version 9.0 (StataCorp LP, College Park, TX, USA). Logistic regression was used to examine the relationship between the outcome variables (duration of illness, hospital admission, and length of stay) with the independent variables discussed below. Multivariate logistic regression models were built using a backwards stepwise approach. We compared categorical variables using the χ^2^ test and continuous variables using the Wilcoxon rank-sum test. A p value <0.05 was considered significant.

### Virus Isolation, Detection, and Identification

Feces samples were cultured for enterovirus by using a human diploid fibroblast cell line. Enterovirus molecular testing was performed directly on CSF samples, dry throat swabs, feces samples, and fecal cell culture supernatants that demonstrated a typical cytopathic effect. In-house seminested reverse transcription–PCR (RT-PCR) was used, which was specific for 2 regions of the 5′ untranslated region (UTR) of the enterovirus genome (*4*,*5*). This 2-region RT-PCR method detects a wide range of enteroviruses. CSF samples were also routinely tested for bacterial pathogens by culture and for herpes simplex virus by PCR.

Enterovirus genotyping was performed by direct sequencing of the viral protein (VP) 1 capsid coding gene. Total RNA was extracted from cell culture supernatant followed by RT-PCR amplification by using primers previously described (*6*). The products were sequenced on both strands by using the ABI Prism BigDye Terminator v3.1 system (Applied Biosystems, Foster City, CA, USA) according to the manufacturer’s instructions. Sequencing reactions were interrogated on an ABI Prism 3130XL 16-channel Genetic Analyzer (Applied Biosystems). The deduced sequence was compared for identification by alignment with enterovirus sequences available in GenBank by using BLASTn (http://blast.ncbi.nlm.nih.gov). The whole viral genome was sequenced by use of primers designed from the obtained sequences and from aligned GenBank E4 sequences. We performed phylogenetic analysis for the VP1 gene using MEGA version 3.0 software (www.megasoftware.net) by the neighbor-joining method with the Kimura 2-parameter model and 1,000 bootstrap replicates.

## Results

We identified 95 cases of acute E4 viral illness. Seventy-six of these cases had a positive PCR CSF result; 8 also had virus detected in throat or feces samples. In the remaining 19, E4 was detected by PCR from a throat swab, a fecal specimen, or both. Records were reviewed on all cases, and interviews were conducted with 48 case-patients or a parent if the case-patient was a child. Interviews were not possible for most indigenous case-patients who lived in remote communities because of a lack of home telephones.

**Figure 1:**
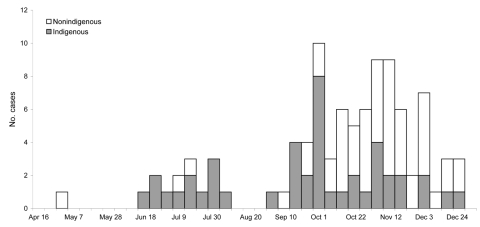
Indigenous and nonindigenous cases of echovirus type 4 virus illness, by week of onset, Northern Territory, Australia, 2007.

Approximately equal numbers of male and female patients were affected (Table 1); most affected children were males (M:F ratio 1.4), and most affected adults were female (M:F ratio 0.8). Forty-six percent of case-patients were indigenous Australians. From the beginning of July through the first week of October, most cases occurred in indigenous infants from remote communities in the Top End; from October through December, most were nonindigenous adults from urban Darwin and nearby communities (Figure 1). The pooled incidence in the 3 main affected regions was 59.1/100,000. Ages of case-patients ranged from 3 days to 56 years (median 12 years), with indigenous case-patients being significantly younger (p<0.0001). Twenty-eight (30%) case-patients were <1 year of age, and 43 (45%) were >15 years of age. Incidence was highest in infants <1 year of age, followed by persons 15–24 years of age.

**Table 1:** Incidence of meningitis caused by echovirus type 4 virus, by age group and sex, Northern Territory, Australia, 2007

Case-patient age group, y	Case-patient sex		Total no. case-patients	Incidence*
	M	F		
<1	16	12	28	1,025.0
1–4	3	3	6	54.9
5–14	11	7	18	71.4
15–24	12	11	23	94.0
25–34	3	10	13	47.3
35–44	2	1	3	11.4
45–64	2	2	4	11.3
>65	0	0	0	0.0
Total	49	46	95	59.1

*Per 100,000 population.

For adults, the most common symptom at onset was headache (70%); for infants and children, it was fever (48%). Common symptoms varied by age group (Table 2). For infants, symptoms were invariably fever and irritability, although for several, poor peripheral perfusion was noted and neonatal bacterial sepsis was clinically suspected. Children and infants were significantly more likely than adults to have fever (p = 0.001); infants were less likely than older children and adults to have vomiting (p<0.0001).

**Table 2:** Prevalence of clinical features and CSF abnormalities in persons affected by echovirus type 4 viral illness, by age group, Northern Territory, Australia, 2007*

Features	No. (%) case-patients by age group, y				Total no. (%) case-patients
	<1	1–4	5–14	>15	
Clinical features	n = 28	n = 6	n = 18	n = 43	N = 95
Fever	28 (100)	6 (100)	17 (94)	32 (74)	83 (87)
Headache	0 (0)	4 (67)	17 (94)	43 (100)	64 (67)
Vomiting	5 (18)	6 (100)	17 (94)	35 (81)	63 (66)
Photophobia†	1 (4)	2 (40)	7 (39)	34 (79)	44 (47)
Diarrhea	16 (57)	0	2 (12)	18 (42)	36 (39)
Neck stiffness	0	1 (17)	9 (50)	21 (51)	31 (33)
Confusion/irritability	16 (57)	2 (33)	2 (11)	7 (17)	27 (29)
Rash	6 (22)	2 (33)	3 (19)	5 (12)	16 (17)
CSF abnormalities	n = 27	n = 5	n = 15	n = 35	n = 82
Glucose <2.7 mmol/L	7 (27)	0	0	3 (9)	10 (12)
Increased CSF protein‡	10 (39)	1 (20)	1 (7)	26 (74)	38 (47)
Leukocyte count >5 cells/mL	8 (30)	5 (100)	15 (100)	31 (90)	59 (72)
Leukocyte count >5 cells/mL and >50% monocytes	5 (19)	2 (40)	10 (67)	28 (80)	45 (55)

*CSF, cerebrospinal fluid. †Presence or absence of clinical features was not discernible in all cases. ‡Age-specific normal values for protein were defined as follows: <1 month, <0.9 mg/L; 1–2 months, <0.77 mg/L; 3 months, 0.6 mg/L; and >3 months, 0.45 mg/L (*7*).

Duration of illness ranged from 3 to 28 days (median 7 days); length of hospital stay ranged from 0 to 10 days (median 3 days). Sequelae, reported for only 4 case-patients, included persistent headache and lethargy up to 4 weeks post onset, but all were mild. Recent similar but milder symptoms, such as fever and headache, were reported for immediate family members in 33 (56%) of 59 case-patients from whom information was available. We did not seek clinical details and investigations of these family members.

Lumbar puncture was performed on 82 case-patients. Fifty-nine (72%) CSF samples showed an increased leukocyte count, 45 (76%) of which had a predominance of mononuclear cells (55% of all samples). Of the 27 infants who had a lumbar puncture, 19 had normal microscopy without pleocytosis, despite all but 1 having the virus detected in the CSF. Initial peripheral leukocyte differential, available for 90 case-patients, was normal for 30 (33%); 50 (55%) had lymphopenia, 21 (23%) had neutrophilia, 2 (2%) had lymphocytosis, and 1 (1%) had neutropenia.

Multivariate analysis showed that of all the potential markers of severity (age, sex, indigenous status, high CSF protein, CSF pleocytosis, lymphopenia, and neutrophilia) only age was associated with admission to hospital and length of stay. The odds ratio of children being admitted to hospital compared with adults was 14.8 (95% confidence interval [CI] 3.2–69.3, p<0.001). When adjusted for indigenous status and sex the odds ratio was 13.0 (95% CI 2.4–69.5, p = 0.003).

The EFVS affected 26 of the 28 communities contacted, and the median attack rate for children was 22% (range 2%–57%); the overall attack rate was 18.6%. Twenty-six cases of viral meningitis occurred in remote indigenous communities in the same area; 19 of these occurred within 3 weeks after the reported onset of the EFVS in their respective communities. Cases of proven viral meningitis in children from remote communities represented 2.0% of the estimated number of EFVS cases in children; however, >50% of these cases came from just 2 communities.

**Figure 2:**
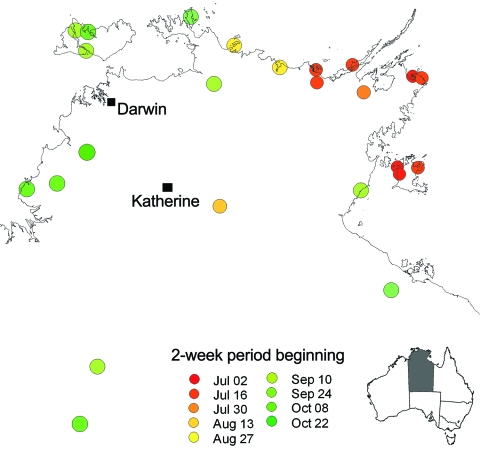
Northern part of the Northern Territory of Australia showing communities affected by the epidemic febrile vomiting syndrome, by week of epidemic peak.

The epidemic began in the east of the northern part of the Northern Territory and progressed to the west over the next 6 months (Figure 2). Nevertheless, the earliest case of E4 meningitis was in April in a Darwin adult, who also reported similar illness in fellow workers.

Not all E4 case-patients exhibited the clinical features of meningitis. For example, 3 adults had neither meningeal symptoms nor CSF pleocytosis, but nevertheless had E4 detected in the CSF or feces. This finding suggests a spectrum of illness typical of enteroviruses, ranging from mild illness to meningitis, and supports the hypothesis that the EVFS was caused by the same virus.

### Molecular Epidemiology

Product from the 5′ UTR PCRs was sequenced from all positive samples and by BLASTn search of the GenBank database; all had the closest homology to the Yanbian strains of echovirus type 4 (AF230973, AF233852). In addition, VP1 capsid coding gene sequences were obtained from 9 of the isolates, and all closely matched the AUS250G strain.

**Figure 3:**
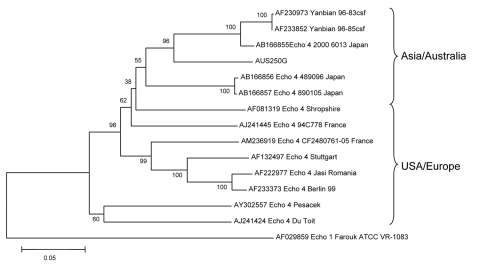
Phylogenetic tree of viral protein (VP) 1 gene sequences showing the relationship of the Australian echovirus type 4 virus (E4) isolate, AUS250G, to E4 strains, 2 Yanbian strains, and an echovirus type 1 sequence. The tree was constructed in MEGA version 3.0 software (www.megasoftware.net) using the neighbor-joining method with the Kimura 2-parameter model and 1,000 bootstrap replicates. Branch numbers represent bootstrap % values. Scale bar represents nucleotide substitutions per site.

A GenBank BLASTn search showed that the whole genome nucleotide sequence of the E4 strain (AUS250G) had 84% homology with the Yanbian strains, and 81% homology with E4 Pesacek (AY302557), a strain isolated in the United States in 1951 (*8*). The AUS250G strain also had a 96% aa homology with the Pesacek strain, which is the only other complete E4 sequence in GenBank. Whole genome amino acid comparisons were not made with the Yanbian strains because amino acid translations of the Yanbian sequences were faulty, presumably due to nucleotide sequence errors. A phylogenetic tree of the complete VP1 nucleotide sequences (Figure 3) shows the relationship of AUS250G to other E4 strains, with the closest matches being a strain isolated in Shiga, Japan (AB166855) and the 2 Yanbian strains. The entire AUS250G genome sequence has been deposited in GenBank under accession no. FJ172447.

## Discussion

This cluster of viral meningitis cases was discovered quickly and was able to be accurately mapped and described because of use of PCR testing of CSF samples. The overlapping symptomatology of both E4 meningitis and EFVS, together with the coincidence of cases of the former with outbreaks of the latter in remote communities and the high incidence of a similar illness in the relatives of meningitis cases, led us to conclude that the EFVS was a milder form of infection with the same virus. Furthermore, E4 was detected in specimens from several patients with fever and vomiting but with no clinical diagnosis of meningitis. The infection appears to have had a high attack rate, particularly in children in remote communities, and ≈2% of case-patients either developed meningitis or were systemically sick enough to be admitted to hospital.

Our estimate of the attack rate must be viewed with caution because we relied on clinic staff estimates of case numbers some weeks after the epidemic rather than through contemporaneous records. Given the time and resource constraints on staff in the remote setting, case numbers could have been overstated. Nevertheless, the EFVS and the high rates of viral meningitis in the community unquestionably stressed the healthcare system, particularly in remote communities. In addition, some schools in urban areas reported several cases of meningitis and absenteeism caused by viral illness in both staff and students, which led to anxiety in the school community. The news media was also interested and published reports of a “brain virus” infecting large numbers of people on the front page of the local newspaper (*9*).

An interesting feature of this study is the number of infants (19/27) whose CSF tested positive for E4 but lacked concomitant pleocytosis. This finding may have been due to our broad case definition and the diligence in investigating febrile neonates to exclude bacterial causes. The immaturity of the immune system in infants who were moderately ill with viremia could also have been a factor.

Previously reported outbreaks of enteroviral meningitis have demonstrated a predominance of males in childhood cases (*10**–**12*), and our study’s findings were consistent with this. Also consistent with previous reports, a significant proportion (30%) of our case-patients were <1 year of age; however, ≈25% of the case-patients in our study were 15–24 years of age, an age group not previously recognized to be at risk.

Most of our cases occurred during July through December, which is late in the dry season of the local subtropical climate. Other reports from regions with temperate climates describe epidemics in the summer and autumn (*10**–**14*).

The spread of the viral illness across the Top End mirrors that reported in Spain in 1991 (*14*). Late in 2007 and early in 2008, spread of the syndrome and enteroviral meningitis into Western Australia and South Australia were reported. Additionally, 116 cases were subsequently laboratory confirmed from Western Australia with all 5′ UTR and 3 VP1 sequences matching the Northern Territory isolates, beginning in the eastern Kimberley region adjacent to the northern part of the Northern Territory and then spreading throughout most of the state. The E4 strain was detected by PCR from the CSF cases of meningitis and from throat swabs from EFVS patients in Western Australia.

From VP1 sequences the E4 strain was most closely related to a Japanese E4 strain and 2 Chinese E4 strains. No further clinical or sequence information is available for the Japanese strain, but the Chinese strains were responsible for a large outbreak of viral meningitis in the Yanbian prefecture of China in 1996 (*2*). Little epidemiologic information is available for this large outbreak, which affected thousands of persons. These authors reported that this outbreak was caused by a new enterovirus isolated from 23 CSF and feces samples. Our review of the whole genome sequence information deposited in GenBank demonstrates that the Yanbian strains and this strain are closely related and belong to the E4 serotype.

This E4 strain is therefore most closely related to other E4 strains from the Western Pacific Rim region. We could not conclude that this E4 enterovirus evolved from the Japanese or Yanbian viruses. However, this E4 strain could have either descended from 1 of these strains or shared a common ancestor virus due to their close VP1 genetic relatedness compared with other E4 strains. The large outbreaks of meningitis caused by both this E4 strain and the Yanbian E4 strains suggest a lack of previous immunity in the populations affected.

We have described an outbreak of human infection with E4 enterovirus in Australia. The high attack rate and the way in which it spread across the Northern Territory and into other states of Australia suggest it may be a newly introduced virus to which the local population has had no previous exposure. The virus likely has been introduced through the northern border where there is considerable human movement between the Northern Territory and the countries to the north. Our findings demonstrate the clinical spectrum of illness and geographic spread of E4 enterovirus infection and add to the knowledge of the molecular epidemiology of echoviruses.
